# The hand grasps the center, while the eyes saccade to the top of novel objects

**DOI:** 10.3389/fpsyg.2015.00633

**Published:** 2015-05-22

**Authors:** Georgiana Juravle, Carlos Velasco, Alejandro Salgado-Montejo, Charles Spence

**Affiliations:** ^1^Department of Systems Neuroscience, University Medical Center Hamburg-Eppendorf, Hamburg, Germany; ^2^Department of Experimental Psychology, University of Oxford, Oxford, UK; ^3^Universidad de La Sabana, Chía, Colombia

**Keywords:** attention, grasping, eye-movements, packaging, indentation

## Abstract

In the present study, we investigated whether indenting the sides of novel objects (e.g., product packaging) would influence where people grasp, and hence focus their gaze, under the assumption that gaze precedes grasping. In Experiment 1, the participants grasped a selection of custom-made objects designed to resemble typical packaging forms with an indentation in the upper, middle, or lower part. In Experiment 2, eye movements were recorded while the participants viewed differently-sized (small, medium, and large) objects with the same three indentation positions tested in Experiment 1, together with a control object lacking any indentation. The results revealed that irrespective of the location of the indentation, the participants tended to grasp the mid-region of the object, with their index finger always positioned slightly above its midpoint. Importantly, the first visual fixation tended to fall in the cap region of the novel object. The participants also fixated for longer in this region. Furthermore, participants saccaded more often, as well saccading more rapidly when directing their gaze to the upper region of the objects that they were required to inspect visually. Taken together, these results therefore suggest that different spatial locations on target objects are of interest to our eyes and hands.

## Introduction

Over the years, a large body of fundamental experimental research has highlighted a number of key behavioral parameters underlying basic reach-to-grasp actions in the laboratory setting. So, for example, when people decide to grasp an object, they typically direct their eyes to it first (Land et al., 1999). Soon thereafter, the hand(s) follow(s) as they reach. Mostly, people end up grasping objects in accordance with their behavioral goals, a process that occurs so automatically in our daily life that we rarely think about it. One relevant question here is whether people grasp and look at novel objects in predictable ways: Do they, for instance, grasp at the location where they fixate initially, or does the hand grasp a position that is different from the the position chosen by the eyes? The answer to this question may provide valuable hints concerning these processes with respect to everyday settings. It also has potentially important implications for ergonomics, marketing, and product development, since the front of product packaging is used to transmit key information to the consumer.

With this consideration in mind, the question that arises is where exactly the eyes are directed to on a specific product/package at the time when consumers are considering whether to choose that product (e.g., see Wedel and Pieters, 2006; Piqueras-Fiszman et al., 2013). This is a key point since product packaging influences consumer behavior through its ability to capture the visual attention of the shopper (Clement, 2007). Supermarkets apparently bring as many as 1000 new products out onto the shelves each month (Nancarrow et al., 1998). Furthermore, research conducted over the last few years has demonstrated that product packaging can be used to enhance the perception of a variety of food and beverage products (Spence and Piqueras-Fiszman, 2012), to help build brand value, and to persuade the consumer to select one brand, or product, over another (Schoormans and Robben, 1997; Rundh, 2009).

Given that consumers frequently pick-up and inspect products before deciding whether to purchase them or not (Piqueras-Fiszman and Spence, 2012; Gallace and Spence, 2014), and given that the location that is grasped is very likely to fall at the object’s center of mass (Blake and Brady, 1992), one might wonder whether visual fixations tend to be focused on the same physical part of product packaging as well. If this were to be the case, then it could be argued that it might make sense to put the most important visual product information at the grasp location for a given product’s packaging. Basic research has shown that humans are very good (i.e., fast, but also accurate) when it comes to estimating the center of mass based on an object’s perceived shape (Goodale et al., 1994). Moreover, not only are center-of-mass-physical-constraints important in choosing the best grasp contact points on an object, but also the observer’s natural grasp angle (Kleinholdermann et al., 2013; Paulun et al., 2014). However, far less research has been conducted on how novel shapes and, in particular, indentations in these target objects, influence grasping and the allocation of visual attention. Introducing an indentation often serves the function of making an object more *graspable*, a quality dependent on both an object’s shape and size. The particular shape of an object will prime a specific (grasping) action from the observer (Tipper et al., 2006). In order for the observer to be able to grasp the object, it needs to have “opposite surfaces separated by a distance less than the span of the hand,” as highlighted by Gibson’s theory of affordances (Gibson, 1979, p. 133).

There is, however, a growing interest in how overt visual attention and eye-movements are directed in order to try and understand how consumers visually explore and attend for a variety of consumer-related areas, here including the design of product packaging (Duchowski, 2002; Wedel and Pieters, 2006). Note that, in recent years, packaging technology has developed rapidly (see Spence and Piqueras-Fiszman, 2012, for a review). Eye-tracking has been proposed as a relevant technique that may help to better understand how the consumer engages with the different elements of visual packaging design (Moskowitz et al., 2009). Having both measures of eye-movements as well as a behavioral measure of visual preference may provide some interesting hints as to what the effect of different packaging designs on consumers’ attention and purchasing behavior may be. It is important to note, though, that research assessing the interplay between eye-movements and grasping in the context of consumer behavior is still scarce.

**FIGURE 1:**
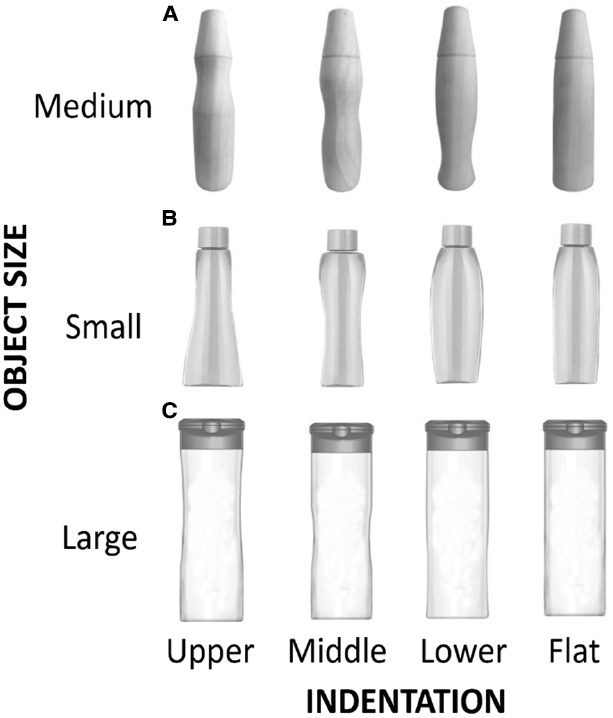
**The left-most three objects shown on the upper row in (A) were utilized for grasping in Experiment 1.** All of the stimuli presented in **(A–C)**, were presented visually in Experiment 2. An indentation could be present in the upper, middle, or lower part of the object, or else the object was presented without indentation.

At present, it is not known if/how the different indentations that one can find nowadays on product packaging influence the way in which a consumer looks at, and grasps, a given product (Pieters and Warlop, 1999; Pieters, 2008; Kamil and Jaafar, 2011; Tonkin et al., 2011). Therefore, two experiments were designed in order to investigate how people grasp and visually inspect novel objects resembling product packaging. In Experiment 1, the focus was on people’s grasping behavior. A set of custom-made wooden objects was designed and machined. The intention was that they should match the shape of a typical deodorant can, the sort that can be found on the shelves of any supermarket. These custom-made objects could either have an upper, middle, or lower indentation. We were interested in using these objects to investigate where the hand would grasp when presented with objects having indentations at different elevations in the vertical plane; see Figure 1A. The hypothesis was that there would be a difference in grasping location as a function of the location of the indentation on the packaging. More specifically, we were interested in the extent to which the indentation *affords* the grasping of the target object (Gibson, 1979), by having participants grasp significantly more often where the indentation is located.

## Experiment 1

### Methods

#### Participants

Fifty-one right-handed participants were recruited to take part in this experiment from the student population of the Pontificia Universidad Javeriana, Bogotá Colombia. All of the participants volunteered and reported normal touch, hearing, as well as normal or corrected-to-normal vision. The experimental session lasted for approximately 10 min. The experiment was reviewed and approved by the Research Committee of the International School of Economics and Administrative Science at Universidad de La Sabana.

#### Apparatus

The participants were seated at a table in a well-illuminated room. The experimenter was seated across the table and placed the objects on the table at the start of each trial. The objects were custom-made from wood, designed so as to match a typical packaging form (see Figure 1A). Three objects (19 cm tall and each weighing 161 g) were used in the study. They differed only in the location of the indentation: lower indentation (3 cm from the bottom of the object), middle indentation (9 cm from the bottom of the object), and upper indentation (12 cm from the bottom of the object). A ruler was drawn on the back of each of the objects. Note that the ruler was only visible to the experimenter.

#### Procedure and Design

The experiment consisted of 45 grasping trials, with 15 trials for each of the object indentations. The order in which the trials were presented was counterbalanced across participants. At the start of each trial, the participants were instructed to close their eyes and wait for the experimenter’s vocal instructions. The experimenter positioned the to-be-grasped object 60 cm from the participant, after which she gave the “Go” signal to start the trial. At the signal from the experimenter, the participants opened their eyes and reached for and grasped the prepared object. The experimenter noted the grasping position and the experiment continued on to the next trial. This grasping location corresponded to the location of the upper limit of the participant’s index finger on the target object.

### Data Analysis and Results

For each of the participants, and for each of the three indentation positions (lower, middle, and upper), the average grasping elevation on the target object was calculated (in cm). A one-way repeated measures analysis of variance (ANOVA) was conducted on the group data with Location of the indentation as the only factor. Mauchly’s test of sphericity was used to ensure that the data did not violate the sphericity assumption. If the assumption was violated, then the Greenhouse–Geisser correction was applied to correct the degrees of freedom; the sphericity violation is reported with ε throughout the text. Any significant main effect was followed-up by means of paired-samples *t*-tests. ${\text{η}}_{\text{p}}^{\text{2}}$ is reported as an effect size estimate for the ANOVA results.

**FIGURE 2:**
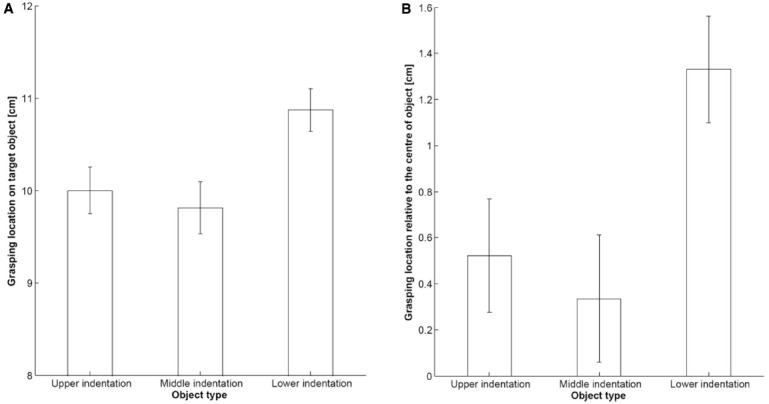
**Main effect of Location of the indentation on the target object for the average grasping location on the target object (A), as well as the grasping differences from the midline of the target object (B), split according to the different indentations (upper, middle, or lower) used in Experiment 1.** The target objects were 19 cm tall. Error bars represent the standard error of the mean.

The results indicated a significant main effect of the Location of the indentation [*F*(2,100) = 11.80; *p* < 0.001; ${\text{η}}_{\text{p}}^{\text{2}}$ = 0.191], with the participants grasping significantly higher if the object had a lower indentation, as compared to an upper (*p* = 0.001), or middle indentation (*p* < 0.001; see Figure 2A). As a second step in the data analysis, grasping differences (GD) were calculated from the midline by subtracting half the product height from each of the final grasping locations. Averages of each of these derived measures were subjected to a repeated-measures ANOVA with the only factor being the location of the indentation. The GD results indicated that the participants always grasped the object slightly above the midline [*F*(2,100) = 10.10; *p* < 0.001; ${\text{η}}_{\text{p}}^{\text{2}}$ = 0.168]: This tendency was more pronounced for the object with a lower indentation (*M* = 1.33 cm GD), than for the object with either an upper (*M* = 0.52 cm GD, *p* = 0.004) or middle indentation (*M* = 0.34 cm GD, *p* < 0.001), with no significant difference between the latter (see Figure 2B).

### Discussion

The results of Experiment 1 clearly demonstrate that when participants are presented with a tall cylindrical novel object with an indentation, they will likely direct their hand toward the upper-middle part, grasping the object slightly above its midpoint. In fact, it would appear as though the lower the indentation, the higher the participants’ grasping location. Although the grasp location did not differ between objects with indentations in the upper and middle positions, it would nevertheless appear numerically as though the participants tended to grasp the novel object in its middle. This result replicates previous results suggesting that grasping is directed toward the center of mass (Blake and Brady, 1992; Goodale et al., 1994; Wing and Lederman, 1998). Such a result therefore supports the hypothesis that the position of the indentation *affords* grasping a cylindrical object.

From an applied perspective, it is of interest to know not only the common grip location on a particular object, but also, where the consumer tends to look when viewing an object, since products often include a variety of visual information (e.g., the brand). Indeed, basic experimental research has already underlined the importance of eye movements, and the control of available visual information for the execution of simple reach-to-grasp movements. For example, an extensive body of laboratory research has revealed that in those situations in which people have to execute a goal-directed movement toward an object of interest, their eyes rapidly move to fixate on the object first (Land et al., 1999). Thereafter, their hand follows toward the fixation location, approximately 100 ms later (Prablanc et al., 1979). Furthermore, when simply asked to view an object, people tend to fixate the object’s center of mass, whereas they fixate around future contact points such as the index finger grasping point location when grasping the object (Brouwer et al., 2009). This preference for fixating the index finger grasping region has recently been explained as dependent on the time to contact with the target object—since the viewer’s index finger tends to be the first digit to make contact with the object when a goal-directed movement is executed (Cavina-Pratesi and Hesse, 2013).

In light of these laboratory findings, we wanted to determine whether the hand and the eye would both land on the middle of an object, or whether instead the hand and eye would have distinct trajectories when apprehending novel object resembling product packaging. At the same time, taking into account such theoretical findings as mentioned above, when one’s interest lies in placing key commercial information on the product packaging, a manufacturer might be keen to know where exactly their customers’ eyes will land first on the target product, or even more, for how long will they linger on certain areas of the product packaging. Additionally, it may be helpful to know which regions of the product benefit from longer fixations. Importantly, here, one has to acknowledge that, in a real-world setting, patterns of grasping are likely to be influenced by the actual design of the packaging. It is for this reason that, in the present study, we were interested in manipulating the *basic* physical attributes needed for a product to attract a consumer’s attention. For example, it has been demonstrated that saccade dynamics will change depending on the size of the image (von Wartburg et al., 2007).

In Experiment 2, we therefore set out to determine the pattern of eye-movements for a selection of objects similar to those used in Experiment 1. For this reason, the participants were presented with pictures of custom-made objects of different sizes, as well as with indentations at different positions. We recorded their eye-movement patterns while they were visually inspecting the object. Our hypothesis was that (various measures of) participants’ eye-movements would differ as a function of the location of the indentation on the object.

## Experiment 2

### Methods

#### Participants

Thirty-four participants took part in Experiment 2. However, five were excluded from the analysis of the data due to the poor quality of their eye movement recordings. The final sample therefore consisted of 29 students. All of the participants reported normal hearing, as well as normal or corrected to normal vision. The experimental session lasted for approximately 30 min. The participants were not paid for taking part in the study.

#### Apparatus

Participants’ eye-movements were recorded by means of an unobtrusive eye-tracker (Tobii TX300, Tobii, Stockholm) with a sampling rate of 300 Hz. The visual stimuli were presented on a 23′ wide TFT Tobii monitor (1920 × 1080 pixels resolution, 60 Hz refresh rate). The experimental stimuli consisted of grayscale photos of the various objects (i.e., the custom-made wooden objects utilized in Experiment 1), or shampoo-like containers, which are also commonly found on the supermarket shelf (see Figure 1, for a depiction of the products used in the present experiment). The objects were small (5.95° × 14.31° visual angle), medium (5.81° × 22.84° visual angle), or large (7.97° × 22.71° visual angle), and they either had an indentation in the upper, middle, or lower part. In the control condition, there was no indentation depicted in the object.

#### Procedure and Design

Object size (small, medium, or large) and Location of the indentation (lower, middle, upper, or absent) were manipulated. The experiment consisted of 12 trials, with the order of presentation of the conditions randomized across participants. Each trial consisted of the presentation of one object from the center of the screen. The participants were instructed to look at the target object for 2.5 s. A fixation cross was presented centrally in between trials.

#### Data Analysis

As a first step in the data analysis, several dependent variables of interest were defined in order to evaluate the visual exploratory behavior of the participants. We were particularly interested in the number of fixations that each participant directed toward each of the objects, the *Fixation count* (i.e., the total number of fixations within a trial), as well as the *Total fixation duration* for each part of the novel objects. Furthermore, for each of the participants, we also derived the total number of fixations that they directed to the object, before reaching the specific region of interest (ROI), *Fixations before ROI*. Lastly, we calculated the time that participants took to make their first fixation on the presented object, *Time to first fixation*.

As a second step, we defined ROIs on the objects, so as to have “landmarks” for data analysis. As such, for each participant, we searched the eye-movement record and decided whether a single fixation fell into one of the following ROIs: the cap of the target object, the upper, middle, or lower region.

Third, we analyzed each of the derived dependent measures (*Fixation count*, *Total fixation duration*, *Fixations before ROI*, and *Time to first fixation*) with repeated measures ANOVAs with the following factors: Object size (small, medium, or large), Indentation location (lower, middle, upper, or absent), and Fixation location (cap, upper, middle, or lower region of the target object). Below, we report the results for all derived dependent measures. The reporting of the results, and their subsequent discussion, is concentrated on the main effects of interest, together with interactions between the Object type and Fixation location variables. Significant two-way interactions observed in the data were followed up with paired-samples *t*-tests; the Holm–Bonferroni correction was utilized in order to control for the family-wise error.

### Results

#### Fixation Count

**FIGURE 3:**
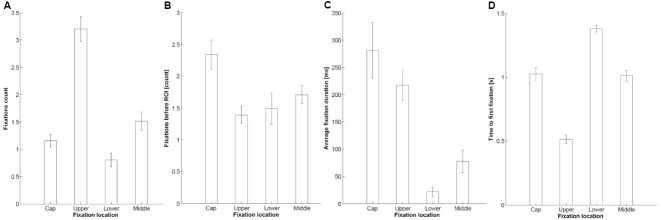
**Main effects of Fixation location for the different dependent measures utilized in Experiment 2: Fixations count (A), Fixations before ROI (B), Average fixation duration (C), and Time to first fixation (D).** Error bars represent the standard error of the mean.

**FIGURE 4:**
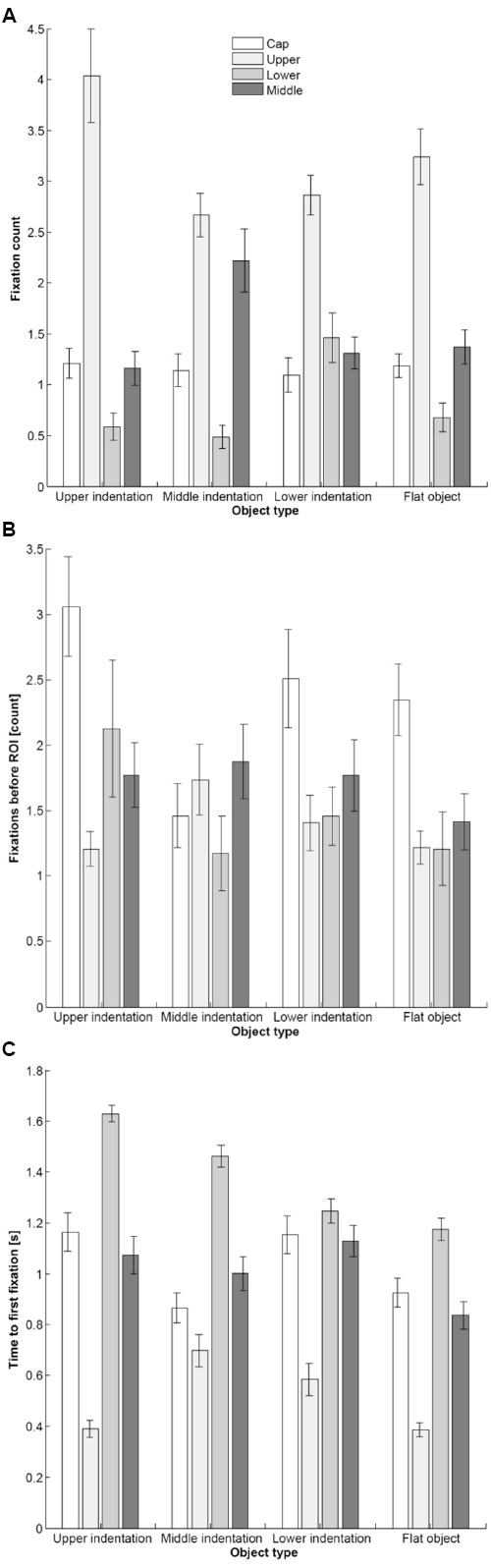
**Depictions of the interaction between Fixation location and Object type for various dependent measures in Experiment 2: (A) Fixations count, (B) Fixations before ROI, and (C) Time to first fixation.** Error bars represent the standard error of the mean.

Analysis of the data revealed that the participants directed significantly fewer fixations to the smaller objects than to either the medium or large objects [main effect of Object size: *F*(2,56) = 10.22; *p* = 0.001; ε = 0.710; ${\text{η}}_{\text{p}}^{\text{2}}$ = 0.267]. Interestingly, the participants directed the majority of their fixations to the upper part of the object, and significantly fewer fixations toward its lower region [main effect of Fixation location: *F*(3,84) = 45.17; *p* < 0.001; ε = 0.752; ${\text{η}}_{\text{p}}^{\text{2}}$ = 0.617; see Figure 3A]. Furthermore, there was a significant interaction between Object size and Fixation location [*F*(6,168) = 3.46; *p* = 0.020; ε = 0.499; ${\text{η}}_{\text{p}}^{\text{2}}$ = 0.110], and a significant three-way interaction between all of the experimental variables [*F*(18,504) = 4.41; *p* = 0.001; ε = 0.293; ${\text{η}}_{\text{p}}^{\text{2}}$ = 0.136]. Importantly, as depicted in Figure 4A, there was an interaction between Object type and Fixation location [*F*(9,252) = 9.66; *p* < 0.001; ε = 0.387; ${\text{η}}_{\text{p}}^{\text{2}}$ = 0.256]. Post hoc tests revealed that this interaction was driven by participants directing significantly more of their fixations toward the upper part of the object when this had an upper indentation, as compared to it having an indentation in the middle (*p* = 0.002). Conversely, the participants directed significantly more of their fixations toward the lower part of the target object when this had a lower indentation as compared to all of the other objects (*p* < 0.001). The participants also directed significantly more of their fixations toward the middle of the object when this had an indentation in the middle, as compared to one in the lower part (*p* = 0.001).

#### Total Fixation Duration

Participants fixated for a significantly longer time on the smaller object, as compared to the medium and large object [giving rise to a main effect of Object size: *F*(2,56) = 3.74; *p* = 0.030; ${\text{η}}_{\text{p}}^{\text{2}}$ = 0.118]. Moreover, for the smaller object, the results indicated that the participants’ gaze lingered for longer in the upper, as compared to the middle region of the object [significant interaction between Object size and Fixation location: *F*(3,84) = 4.46; *p* = 0.006; ε = 0.494; ${\text{η}}_{\text{p}}^{\text{2}}$ = 0.137]. Lastly, the most important result to emerge from this analysis was that overall, the participants looked for significantly longer at the cap of the object [main effect of Fixation location: *F*(3,84) = 14.86; *p* < 0.001; ε = 0.514; ${\text{η}}_{\text{p}}^{\text{2}}$ = 0.374; see Figure 3C]. This result implies that in the absence of any logo, such as was the case in the present study, participants may exhibit an intrinsic preference for the upper cap region of an object they see for the first time.

#### Fixations before ROI

Participants made significantly more fixations before their eyes landed on the ROI when viewing the larger, as compared to the smaller, objects [main effect of Object size: *F*(2,56) = 6.13; *p* = 0.004; ${\text{η}}_{\text{p}}^{\text{2}}$ = 0.180]. Moreover, the main effect of Fixation location [*F*(3,84) = 7.34; *p* < 0.001; ${\text{η}}_{\text{p}}^{\text{2}}$ = 0.208] indicated that the participants’ gaze tended to linger for longer in the cap region, than on any other part of the experimental packaging. That is, the participants started by looking at the very top of the objects, before fixating on a particular ROI (see Figure 3B). Lastly, an interaction between Object type and Fixation location [*F*(9,252) = 3.33; *p* = 0.005; ε = 0.627; ${\text{η}}_{\text{p}}^{\text{2}}$ = 0.106], indicated that for all of the novel objects bar those having an indentation in the middle, the pattern of eye movements involved significantly more fixations before ROI for the region around the cap of the target object (see Figure 4B).

#### Time to First Fixation

The time to first fixation data indicated significant main effects of all manipulated variables: Object size [*F*(2,56) = 3.55; *p* = 0.035; ${\text{η}}_{\text{p}}^{\text{2}}$ = 0.112], Object type [*F*(3,84) = 26.98; *p* < 0.001; ε = 0.759; ${\text{η}}_{\text{p}}^{\text{2}}$ = 0.491], and Fixation location [*F*(3,84) = 80.62; *p* < 0.001; ε = 0.752; ${\text{η}}_{\text{p}}^{\text{2}}$ = 0.742]. The participants took significantly less time to fixate on the medium-sized objects, as well as taking significantly less time to fixate on the objects that did not have an indentation. Furthermore, the participants initiated their first saccade toward the upper region of the target object significantly faster than to any of the other regions. If the first saccade was directed toward the lower region of the target object, it took participants significantly longer to initiate it, as compared to any of the other regions (see Figure 3D).

The time to first fixation data also accommodated significant two-way interactions between Object size and Object type [*F*(6,168) = 6.28; *p* < 0.001; ${\text{η}}_{\text{p}}^{\text{2}}$ = 0.183], Object size and Fixation location [*F*(6,168) = 5.55; *p* < 0.001; ${\text{η}}_{\text{p}}^{\text{2}}$ = 0.165], and a significant three-way interaction [*F*(18,504) = 8.68; *p* < 0.001; ε = 0.532; ${\text{η}}_{\text{p}}^{\text{2}}$ = 0.237]. We were particularly interested in the interaction between Object type and Fixation location [*F*(9,252) = 8.21; *p* < 0.001; ε = 0.679; ${\text{η}}_{\text{p}}^{\text{2}}$ = 0.227; see Figure 4C]. Post hoc tests revealed that this interaction resulted from a series of significant differences: That is, the amount of time required to direct the first fixation to the cap of a target object was shorter for those objects with an upper indentation, as compared to an indentation in the middle (*p* = 0.001), and those having no indentation at all (*p* = 0.001). Interestingly, however, our participants saccaded significantly more rapidly to the cap region for objects with a middle indentation, as compared to objects with a lower indentation (*p* = 0.001). Along similar lines, participants directed their first fixation to the upper region of the target object if this had a middle, as compared to an upper (*p* < 0.001), or lower indentation (*p* < 0.001). These results therefore indicate that only when we compare the unindented object with the object with a lower-indentation were participants faster to direct their first fixation in the upper region (*p* < 0.001).

### Discussion

The results of Experiment 2 clearly demonstrate that the cap of novel objects, as well as the upper region of such experimentally-created objects, receives preferential visual processing (i.e., overt attention). The most important result to emerge from the analysis of the data of our second experiment was that the participants inspected the tested objects’ cap region for the *longest* time. In addition, they saccaded *more often*, as well as their saccades were *fastest* when directed to the upper region of the objects that they were required to inspect visually. Moreover, it would also appear that the visual scanning of a novel object begins, most of the time at least, in the same cap region, a result that argues against a center bias (Tatler, 2007; Tseng et al., 2009). This was the case for all the novel object formats tested in Experiment 2, except for the object with an indentation in the middle.

## General Discussion

The two experiments reported in the present study were separately designed to determine where the hand grasps (Experiment 1), and where the eyes fixate (Experiment 2) when people are faced with a novel object. In order to address this question, the participants in our first experiment grasped a prototypical object having either an upper, middle, or lower indentation. The results of Experiment 1 revealed that the lower the indentation, the higher the grasp. Nevertheless, irrespective of the location of the indentation, grasping tended to cluster around the midpoint of the object. In Experiment 2, we went on to investigate whether the grasping location for such novel objects would match the location where the participant’s eyes tended to fixate. On the basis of previous laboratory research, we already know that when preparing a reach-to-grasp action, the eyes first “fly” to the goal location, after which time the hand follows to the saccade location fairly rapidly (Prablanc et al., 1979). The results of Experiment 2 indicated that amongst the tested novel objects the upper/cap region seems to be the preferential target for the eyes to land on: That is, the participants first fixate within the cap region. Furthermore, they also tend to fixate for longer in this region. At the same time, participants direct most of their fixations to the upper region of novel objects tested here, and they take the shortest time to saccade to the same region.

A word of caution is perhaps in order here though: It is important to note that whereas the participants in Experiment 1 grasped the target products that were presented in front of them, in Experiment 2, they were only presented with 2D photos of the target packages. We recorded/derived eye-movement patterns for these photos, and not for the real objects. Importantly, though, people nowadays will very often first be exposed to the 2D image of a product they are interested in before they actually touch/grasp it (e.g., just think of on-line shopping, printed media, or advertising).

On such grounds, it could therefore be argued that our eye-movement results should, in the future, be replicated with participants simultaneously grasping real products, in order to fully understand what is going on. One should not, however, underestimate the technical challenges associated with such simultaneous monitoring of a participant’s gaze and grasp, although some recent advances have undoubtedly been made in this direction (Deng et al., 2014). Note that when comparing eye-movements while viewing 2D photos of natural images, to the same photos with added 3D disparity information, participants make significantly more fixations, as well as the length of their saccades significantly shortens while inspecting the latter. Crucially, their total fixation duration is comparable between the 2D/3D renditions (Jansen et al., 2009). Even more, if when inspecting an object of interest in a 2D natural scene we usually utilize small saccades to trace its outline (Yarbus, 1967; Hinger et al., 2009), these saccades become shorter and faster with 3D images, but image saliency is nevertheless still comparable for images with or without disparity information (Jansen et al., 2009).

Having underlined such a shortcoming for the experiments reported here, it is nevertheless important to stress that our results indicate that, as one might have expected, the hand does indeed roughly grasp the location of the indentation, whereas the eyes are directed more toward the cap region of the custom-made target objects presented in Experiment 1 (see Figure 1A). Our results therefore indicate that different spatial locations on target objects are of interest to our eyes and hands. In light of these findings, a key question to address in future research is whether a manufacturer would benefit from introducing an indentation in their traditionally straight-sided product packaging. The putative reason for doing this might be to more narrowly focus the consumer’s visual attention on a specific region of the product packaging. Note that earlier studies have demonstrated the facilitatory effects of visual attention at the goal location of a saccade (Paillard, 1982), as well the crucial role of visual information available at the beginning of a goal-directed movement (Binsted et al., 2001; Fukui and Inui, 2006). It has been shown that we tend not to direct our eyes toward locations on the moving hand or on the trajectory to the object of interest, but rather, we fixate primarily on the key locations for manipulating the target object (Johansson et al., 2001). That is, when the current goal is simply to view the objects, we will most likely fixate at the center of mass. However, when we intend to reach and grasp for a certain object of interest, we will tend to fixate at the future grasp contact points (Brouwer et al., 2009; see also Land and Hayhoe, 2001, for an elegant experimental investigation of eye-movements distribution during everyday activities, and Causer et al., 2013, for a review).

On a different note, one needs to acknowledge the contribution of the various sensory systems that contribute to goal-directed action (Driver and Spence, 1998; Atkins et al., 2001; Talsma et al., 2010). It would seem though that vision absorbs the majority of sensory processing capacity during the execution of the movement (Brozzoli et al., 2009), since deficits in the other senses are reported during the execution period of a goal-directed action (e.g., tactile sensation is suppressed, Juravle et al., 2010; see also Gallace and Spence, 2014). The preparatory phase, or the intention deriving phase, as well as the post-movement phase appear though to benefit from enhancement in perceptual tasks. For example, higher visual acuity has been demonstrated at the goal location of an upcoming saccade (Deubel and Schneider, 1996), as well as that of a prehensile movement (Schiegg et al., 2003), even before the actual saccade or hand movement has been initiated.

Our results also indicate that participants fixate for longer on the cap region of the novel object. Such an outcome could be useful in terms of providing guidelines as to where the most important messaging should be placed: One practical suggestion to emerge from the research reported here is that the cap of the product appears to be particularly relevant in this regard, whereas the middle region could on the other hand be spared of visual content and perhaps be designed for a better grip. The lower part of cylindrical packaging could, in turn, carry the less important messages for a first glance (i.e., the list of ingredients). Furthermore, with this consideration in mind, an interesting follow-up question would be where to place a product on a supermarket shelf, given that either top, middle, or the lower region is more easily visually made available to the customer. At the same time, marketers might need to consider, at the stage of designing a product, their preferential product positioning on the supermarket shelf, with the product’s physical attributes engineered according to the intended product location.

Finally, it is perhaps worth dwelling for a moment on the focus of visual attention (Scholl, 2001) on the caps of the various products documented in our Experiment 2. Because we were interested in having a baseline measure of eye-movements toward given products, the participants in this experiment viewed pictures of simple plain objects without any type of logo, or anything that could be taken for a promotional message. With this consideration in mind, one could ask whether similar results would have been obtained if one were to utilize logos, or product related messages on the entire surface of a product. For example, recent eye-movement research has highlighted that consumers prefer the logos in the upper left corner of packaging they are presented with (Rebollar et al., 2014). A corollary of this question would then regard the type of visual processing responsible for eye-preference for a product’s cap region, such as we find in the current study. Note that our study suggests a top-down (intrinsic) preference for the upper/cap region of certain (e.g., tall cylindrical) products. According to our findings, placing very salient logos at any other location on the target products should result in significant slower times to saccade to, significantly fewer, as well as shorter saccades, as compared to logos placed on the upper/cap region. Future experiments will be needed in order to investigate which type of visual orienting is responsible for product selection in the supermarket as well as how such basic visual orienting interacts with a viewer’s current goals and interests.

### Conflict of Interest Statement

The authors declare that the research was conducted in the absence of any commercial or financial relationships that could be construed as a potential conflict of interest.
